# Astaxanthin Exerts Immunomodulatory Effect by Regulating SDH-HIF-1α Axis and Reprogramming Mitochondrial Metabolism in LPS-Stimulated RAW264.7 Cells

**DOI:** 10.3390/md20110660

**Published:** 2022-10-25

**Authors:** Luchuanyang Sun, Sangeun Kim, Ryoichi Mori, Nobuyuki Miyaji, Takeshi Nikawa, Katsuya Hirasaka

**Affiliations:** 1 Graduate School of Fisheries and Environmental Sciences, Nagasaki University, Nagasaki 8528521, Japan; 2 Department of Pathology, Graduate School of Biomedical Sciences, Nagasaki University, 1-12-4 Sakamoto, Nagasaki 8528523, Japan; 3 Toyo Koso Kagaku Co., Ltd., Chiba 2790041, Japan; 4 Department of Nutritional Physiology, Institute of Medical Nutrition, Tokushima University Medical School, Tokushima 7708503, Japan; 5 Organization for Marine Science and Technology, Nagasaki University, Nagasaki 8528521, Japan

**Keywords:** astaxanthin, mitochondrial ROS, mitochondrial energy metabolism, mitochondrial succinate dehydrogenase, hypoxia-inducible factor-1α, interleukin-1β

## Abstract

Astaxanthin (AX) is a carotenoid that exerts potent antioxidant activity and acts in cell membranes and mitochondria, which consist of the bilayer molecules. Targeting mitochondria to ameliorate inflammatory diseases by regulating mitochondrial metabolism has become possible and topical. Although AX has been shown to have anti-inflammatory effects in various cells, the mechanisms are quite different. In particular, the role of AX on mitochondrial metabolism in macrophages is still unknown. In this study, we investigated the effect of AX on mitochondria-mediated inflammation and its mechanisms in lipopolysaccharide (LPS)-stimulated RAW264.7 cells. AX attenuated the mitochondrial O_2_^−^ production and maintained the mitochondrial membrane potential, implying that AX preserved mitochondrial homeostasis to avoid LPS stimulation-induced mitochondrial dysfunction. Additionally, AX prevented the decrease in mitochondrial complexes I, II, and III, which were caused by LPS stimulation. Especially, AX inhibited the reduction in mitochondrial succinate dehydrogenase (SDH; complex II) activity and upregulated the protein and mRNA level of SDH complex, subunit B. Furthermore, AX blocked the IL-1β expression by regulating the SDH-HIF-1α axis and suppressed the energy shift from an OXPHOS phenotype to a glycolysis phenotype. These findings revealed important effects of AX on mitochondrial enzymes as well as on mitochondrial energy metabolism in the immune response. In addition, these raised the possibility that AX plays an important role in other diseases caused by SDH mutation and metabolic disorders.

## 1. Introduction

The immune system is an important system for the body to trigger immune responses and perform the necessary functions. The immune system consists of organs (bone marrow, spleen, lymph nodes, etc.), immune cells (phagocytes, lymphocytes, etc.), and immunologically active substances (lysozyme, leukocytes interferon, interleukin, etc.). Macrophages, a member of phagocytes, are allosteric, and the variations of their phenotype and function are influenced by different factors, especially by the surrounding microenvironment [1,2]. Commonly, macrophages are polarized into different subtypes, such as M1 and M2 macrophages, depending on the changes in the environment. M1 (classical) macrophages (e.g., lipopolysaccharide (LPS) stimulation) usually exhibit a pro-inflammatory response and release large amounts of pro-inflammatory cytokines, such as tumor necrosis factor (TNF)-α, interleukin (IL)-1β, inducible nitric oxide synthase (iNOS), and monocyte chemoattractant protein (MCP)-1. On the other hand, M2 (alternative) macrophages (e.g., IL-4/IL-13 stimulation) usually exhibit an anti-inflammatory response and repair function [3]. In infected tissues or organs, macrophages are primarily polarized to the M1 phenotype, displaying the pro-inflammatory function to help the host resist pathogens. Subsequently, in order to avoid the excessive damage of pro-inflammatory factors to the host, macrophages are polarized to the M2 phenotype to drive an anti-inflammatory response and repair damaged tissues or organs [2]. Thus, modulation of immune function is carried out by regulating macrophage polarization, leading to maintained homeostasis.

Mitochondria have long been known as energy factories. Metabolites are oxidized through the tricarboxylic acid (TCA) cycle in the mitochondrial matrix and then pass through the electron transport chain (ETC) in the inner membrane of mitochondria to produce adenosine-triphosphate (ATP). Recently, mitochondria have been considered an energy source for immunity in addition to being the powerhouses of the cell [4]. With the special status of mitochondria as the central hub of metabolism, they are necessary to maintain and establish immune cell phenotypes [5]. Similarly, mitochondrial metabolism modulates the biological activity and functions of macrophages [6]. The modification of mitochondrial metabolism and physiological function, such as mitochondrial reactive species (mtROS), mitochondrial membrane potential (MMP), mitochondrial DNA (mtDNA), mitochondrial oxidative phosphorylation (OXPHOS), TCA cycle, and mitochondrial ultrastructure, are crucial indicators of macrophage activation and polarization [7,8,9,10]. In addition, the remodeling of mitochondrial metabolism in macrophages is regarded as an anti-inflammatory signal [11]. Recently, targeting mitochondria to ameliorate inflammatory diseases by regulating mitochondrial metabolism has become possible.

**Figure 1:**
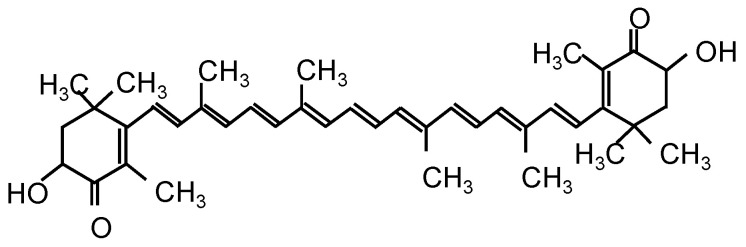
Structure of Astaxanthin.

Astaxanthin (AX; 3,3′-dihydroxy-β, β′-carotene-4,4′-dione) is widely known as a potent antioxidant. It is a kind of carotenoid with long conjugated double bonds, keto groups, and hydroxyl groups (as shown in Figure 1), and naturally accumulates in microalgae, yeast, crustaceans, fish epidermis, and other biologicals [12,13]. The unique molecular structure of AX allows for its insertion through the lipid bimolecular layer of cell membranes, leading to stronger protection and free-radical-scavenging effects at the cell membrane than other antioxidants such as β-carotene, α-tocopherol, and vitamin C [13]. In recent years, AX has been studied for mitochondria-related diseases due to its unique structure and activity. AX reportedly maintains mitochondrial integrity by reducing oxidative stress, prevents the loss of mitochondrial membrane potential, and increases mitochondrial oxygen consumption, which inhibits mitochondrial dysfunction [14,15,16]. In our previous study, we also found that AX was easily accumulated in the mitochondria of muscle cells rather than in the cytosol and prevented mitochondrial disorder-induced muscle atrophy by regulating mitochondrial function [17]. Moreover, not only due to its antioxidant activity, but AX’s anti-inflammatory activity also plays a role in many chronic and acute diseases such as neurological diseases, diabetes, gastrointestinal diseases, hepatic and renal diseases, and skin and eye disorders [18]. Although the mechanisms of anti-inflammatory activity of AX have been partially investigated, a large part of the mechanisms is still unknown. In addition, crucially, the details of many of the identified anti-inflammatory mechanisms and whether there are some cross-talks between different signaling pathways still deserve further exploration. Hence, we investigated the anti-inflammatory effects of AX in LPS-stimulated RAW264.7 cells and the mechanisms of AX acting on mitochondrial metabolism under the M1 macrophages. We identified a specific effect of AX on succinate dehydrogenase (SDH, an important enzyme on mitochondria) and proposed that there is a regulatory effect on the SDH-HIF-1α axis of AX, which is partially involved in the anti-inflammatory mechanisms of AX. Remarkably, AX reprogrammed mitochondrial metabolism and suppressed a shift from an OXPHOS phenotype to a glycolytic phenotype during M1 macrophage polarization.

## 2. Results

### 2.1. AX Attenuates the mRNA Expression and Secretion of IL-1β in LPS-Stimulated RAW264.7 Cells

**Figure 2:**
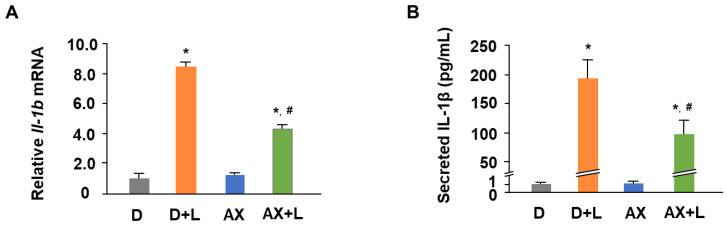
Astaxanthin attenuates the mRNA expression and secretion of IL-1β in LPS-stimulated RAW264.7 cells. RAW264.7 cells were in the presence or absence of AX (10 μM) before being stimulated with the LPS (1 μg/mL)/PBS (-) for 6 h (**A**) or 24 h (**B**). (**A**) qRT-PCR analysis of *Il-1b* mRNA level. The total RNA of the cells was extracted and subjected to qRT-PCR. The expression ratio is relative to that of *18S*. (**B**) ELISA analysis of IL-1β secreted level. Supernatants of the medium were collected for ELISA detection. Data are represented as mean ± S.D. (A, *n* = 6; B, *n* = 3). * *p* < 0.05, compared with D and AX groups. # *p* < 0.05, compared with D + L group. D, DMSO + PBS (-) group; D + L, DMSO + LPS group; AX, Astaxanthin + PBS (-) group; AX + LPS, Astaxanthin + LPS group.

Before all analysis of the items, a CCK-8 assay was conducted to confirm the condition of the cells. The added astaxanthin did not affect cell viability, then we continued to analyze other items. The effect of AX on IL-1β mRNA expression and secretion is shown in Figure 2. LPS, a gram-negative bacterial product, infects cells and induces inflammation, which is characterized by the release of pro-inflammatory cytokines such as IL-1β, TNF-α, and IL-6. AX has been known to have an anti-inflammatory effect in vivo and in vitro. However, the modulation of different pro-inflammatory cytokines also implies different mechanisms involved in anti-inflammation. To investigate the influence of AX on LPS-stimulated RAW264.7 cells, we measured the mRNA expression of pro-inflammatory cytokines by qRT-PCR and secretion of pro-inflammatory cytokines by ELISA. Consistent with previous studies [19,20], the mRNA expression and secretion of IL-1β, TNF-α, and IL-6 were increased significantly due to the stimulation of LPS (Figure 2A; TNF-α, IL-6 data not shown; IL-1β, 8-fold, compared to D). On the contrary, AX downregulated the expression and secretion of these pro-inflammatory cytokines, especially in IL-1β (Figure 2B; TNF-α, IL-6 data not shown; IL-1β, 180-fold, compared to D). These results displayed the anti-inflammatory effect of AX, suggesting that AX may play an anti-inflammatory role by inhibiting IL-1β through a specific signaling pathway.

### 2.2. AX Alleviates Mitochondrial O_2_^−^ Production and Maintains MMP in LPS-Stimulated RAW264.7 Cells 

**Figure 3:**
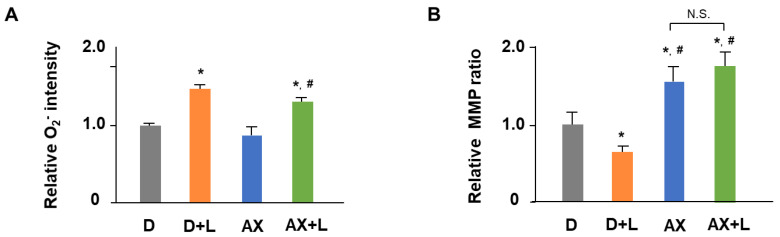
Astaxanthin alleviates mitochondrial O_2_^−^ production and maintains MMP in LPS-stimulated RAW264.7 cells. RAW264.7 cells were in the presence or absence of AX (10 μM) before being stimulated with the LPS (1 μg/mL)/PBS (-) for 6 h. (**A**) MitoSOX detection of mitochondrial O_2_^−^ production. Data are represented as mean ± S.D. (*n* = 6). * *p* < 0.05, compared with D and AX groups. # *p* < 0.05, compared with D + L group. (**B**) JC-1 dye detection of MMP. Data are represented as mean ± S.D. (*n* = 6). * *p* < 0.05, compared with D group. # *p* < 0.05, compared with D + L group. N.S., not significant. D, DMSO + PBS (-) group; D + L, DMSO + LPS group; AX, Astaxanthin + PBS (-) group; AX + LPS, Astaxanthin + LPS group.

The effect of AX on mt ROS and MMP is shown in Figure 3. Excessive superoxide production by mitochondria is usually thought to be a partial source of inflammation and a catalyst for accelerated inflammation. The excessive ROS production is accompanied by mitochondrial dysfunction, which features a decrease in MMP, disturbance of mitochondrial respiration complex, and so on. In this study, to investigate whether the anti-inflammatory effect of AX is associated with mitochondrial function, we first analyzed the effect of AX on mitochondrial O_2_^−^ production and MMP by Mito SOX and JC-1 dye, respectively. Mitochondrial O_2_^−^ production in the LPS stimulated group (D + L) was significantly increased by approximately 1.7-fold, compared with that in the control group (D). In contrast, the addition of AX alleviated mitochondrial O_2_^−^ production in the AX + LPS group (Figure 3A). Likewise, MMP in D + L was significantly decreased by 30%, compared with that in D. Treatment of AX prevented the decrease in MMP due to LPS stimulation and maintained higher MMP levels (Figure 3B). These results revealed that LPS-induced inflammation is associated with the alteration of mitochondrial O_2_^−^ production and MMP, while AX played a regulatory role in mitochondrial O_2_^−^ production and MMP in LPS-stimulated RAW264.7 cells.

### 2.3. AX Prevents the Reduction of SDH Activity and Upregulates the Protein and mRNA Level of Succinate Dehydrogenase Subunit B (SDHB) in LPS-Stimulated RAW264.7 Cells

**Figure 4:**
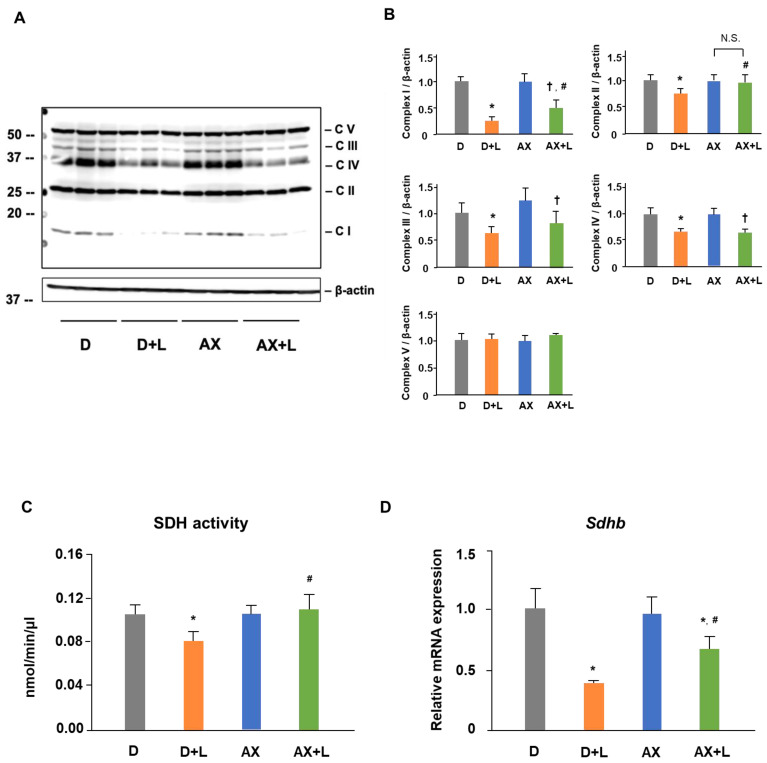
Astaxanthin prevents the reduction in SDH activity and up-regulates the protein and mRNA level of SDHB in LPS-stimulated RAW 264.7 cells. RAW 264.7 cells were in the presence or absence of AX (10 μM) before being stimulated with the LPS (1 μg/mL)/PBS (-) for 6 h (**D**) or 24 h (**A**,**C**). (**A**) Western blot analysis of total OXPHOS proteins and β-actin. (**B**) Quantification of (**A**). NADH dehydrogenase beta subcomplex subunit 8 of Complex I (NDUFB8), succinate dehydrogenase subunit B of Complex II (SDHB), cytochrome b-c1 complex subunit 2 of Complex III (UQCRC2), cytochrome c oxidase subunit 1 of Complex IV (MTCO1), and ATP synthase subunit alpha of Complex V (ATP5A). Data are represented as mean ± S.D. (**A** and **B**, *n* = 3) * *p* < 0.05, compared with the D group. ^†^ *p* < 0.05, compared with AX group. # *p* < 0.05, compared with D + L group. N.S., not significant. (**C**) Succinate dehydrogenase (SDH) activity assay. (**D**) *Sdhb* mRNA expression. The expression ratio is relative to that of *18S*. Data are represented as mean ± S.D. ((**C**), *n* = 4; (**D**), *n* = 6). * *p* < 0.05, compared with D and AX groups. # *p* < 0.05, compared with D + L group. N.S., not significant. D, DMSO + PBS (-) group; D + L, DMSO + LPS group; AX, Astaxanthin + PBS (-) group; AX + LPS, Astaxanthin + LPS group.

The effect of AX on SDH activity, SDHB protein level, and *Sdhb* mRNA level are shown in Figure 4. Mitochondrial respiratory complexes play a vital role in mitochondrial function. Meanwhile, changes in the phenotype, function and even energy metabolism of macrophages are accompanied by the variation of mitochondrial respiratory complexes in a certain sense. To further study the effect of AX on mitochondria in LPS-stimulated RAW264.7 cells, we examined the alteration of mitochondrial respiratory complexes by immunoblotting. The amounts of mitochondrial respiratory complexes I, II, III, and IV proteins in the D + L group were significantly decreased (I, 70% decreased; II, 30% decreased; III, 45% decreased; IV, 30% decreased), compared with those in the D group. Interestingly, we found that AX prevented the reduction in mitochondrial respiratory complexes I, II, and III, but not IV, which was caused by LPS stimulation (Figure 4A,B). In particular, mitochondrial respiratory complex II, succinate dehydrogenase subunit B (SDHB), is an important complex protein on the mitochondrial electron transport chain (ETC) as well as an essential enzyme in the tricarboxylic acid cycle (TCA cycle), as it is the connection point of ETC and TCA cycle, and critical for the inflammatory response. Based on these results, we continued to investigate the effect of AX on the activity and the mRNA expression of SDH. In accordance with the findings of protein expression, AX prevented the reduction in SDH activity (Figure 4C). Furthermore, treatment of AX improved the decrease in *Sdhb* mRNA expression by LPS stimulation (Figure 4D). These results demonstrated that AX has a regulatory effect on mitochondrial respiratory complex proteins in LPS-stimulated RAW264.7 cells.

### 2.4. AX Blocks the IL-1β Expression by Regulating SDH-HIF-1α Axis in LPS-Stimulated RAW264.7 Cells

**Figure 5:**
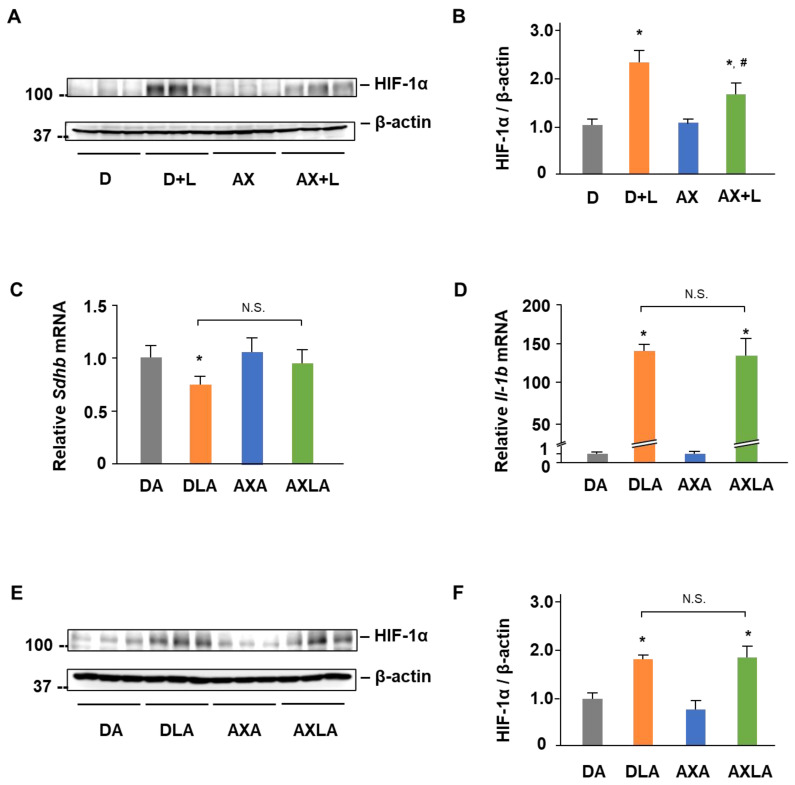
Astaxanthin blocks the IL-1β expression by regulating SDH- HIF-1α axis in LPS-stimulated RAW264.7 cells. (**A**) Western blot analysis of HIF-1α and β-actin. RAW264.7 cells were in the presence or absence of AX (10 μM) before being stimulated with the LPS (1 μg/mL)/PBS (-) for 24 h. (**B**) Quantification of (**A**). (**C**) *Sdhb* mRNA expression. (**D**) *Il-1b* mRNA expression. The expression ratio is relative to that of *18S*. (**E**) Western blot analysis of HIF-1α and β-actin. (**F**) Quantification of (**E**). (**C**–**F**) RAW264.7 cells were in the presence or absence of AX (10 μM) before being stimulated with LPS (1 μg/mL)/PBS (-) for 3 h (**C**,**D**) or 20 h (**E**,**F**), then atpenin A5 (AA5) was added and treated for a further 4 h together with LPS. Data are represented as mean ± S.D. ((**B**), *n* = 3; (**C**,**D**), *n* = 6; (**F**), *n* = 3). * *p* < 0.05, compared with D and AX groups. # *p* < 0.05, compared with D + L group. N.S., not significant. D, DMSO + PBS (-) group; D + L, DMSO + LPS group; DA, DMSO + AA5 group; DLA, DMSO + LPS + AA5 group; AX, Astaxanthin + PBS (-) group; AX + LPS, Astaxanthin + LPS group; AXA, AX + AA5 group; AXLA, AX + LPS + AA5 group.

The effect of AX on HIF-1α induced IL-1β is shown in Figure 5. During classical M1 macrophage activation, alterations in certain mitochondrial metabolites are regarded as an inflammatory signal-inducing cytokine (such as IL-1β) released through HIF-1α [21]. To investigate whether HIF-1α plays a role in LPS-stimulated RAW264.7 cells and the effect of AX on the HIF-1α, we detected the protein expression of HIF-1α in LPS-stimulated RAW264.7 cells. Interestingly, the HIF-1α protein level was significantly upregulated 2.2-fold by LPS (Figure 5A,B). In contrast, AX downregulated the HIF-1α protein expression caused by LPS stimulation. Subsequently, to confirm whether AX affects the mitochondria-mediated HIF-1α signaling pathway, we used Atpenin A5 (AA5), a kind of inhibitor of SDH. Although the co-treatment of LPS and AA5 significantly decreased the *Sdhb* mRNA level, AX pretreatment did not improve its expression completely (Figure 5C). Moreover, the addition of AA5 abrogated the original downregulation of IL-1β (Figure 5D) and HIF-1α (Figure 5E,F) by AX. These results demonstrated that AX blocked the IL-1β expression by regulating the SDH-HIF-1α axis.

### 2.5. AX Suppresses a Shift from an OXPHOS Phenotype towards a Glycolytic Phenotype in LPS-Stimulated RAW264.7 Cells

**Figure 6:**
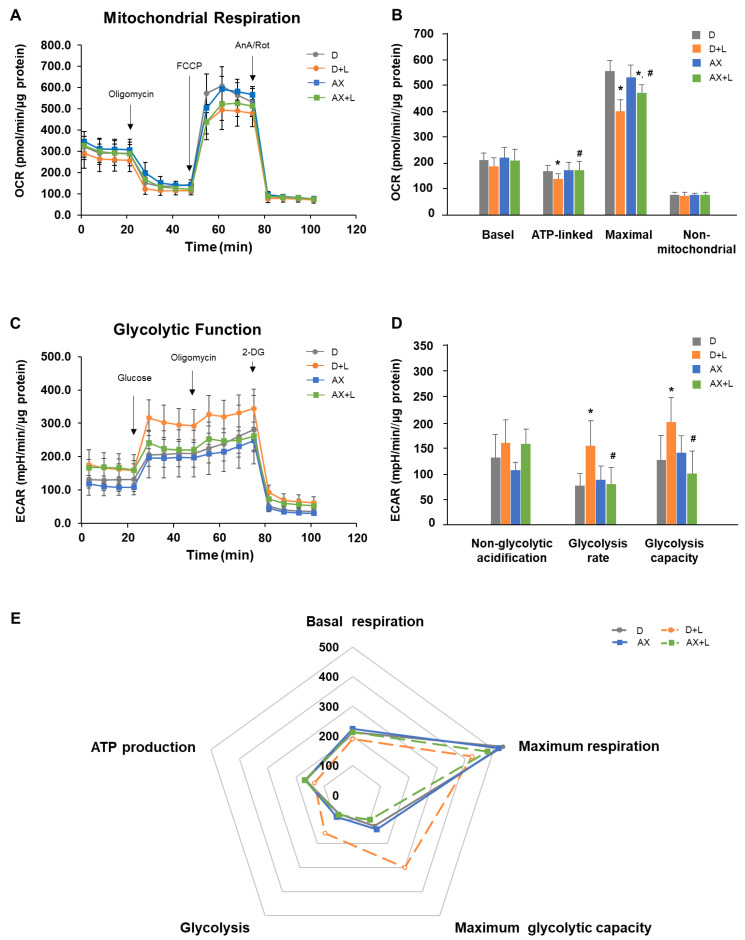
Astaxanthin suppresses a shift from an OXPHOS phenotype towards a glycolytic phenotype in LPS-stimulated RAW264.7 cells. RAW264.7 cells were in the presence or absence of AX (10 μM) before being stimulated with the LPS (1 μg/mL)/PBS (-) for 3 h. During extracellular flux analysis, cells were treated with (**A**) oligomycin, FCCP, AnA/Rot to detect the mitochondrial respiration according to the OCRs levels or treated with (**C**) glucose, oligomycin, 2-DG to detect the glycolytic function according to the ECARs levels. (**B**) Basal respiration, mitochondrial ATP-linked respiration, maximum respiration, and non-mitochondrial respiration were calculated based on OCRs levels. (**D**) Non-glycolytic acidification, glycolysis rate, and maximum glycolysis capacity were calculated based on ECARs. (**E**) Both calculated OXPHOS and glycolysis metabolic parameters were displayed in the summary. Data are represented as mean ± S.D. (*n* ≥ 12). * *p* < 0.05, compared with D and AX groups. # *p* < 0.05, compared with D + L group. FCCP, carbonyl cyanide-4 (trifluoromethoxy) phenylhydrazone; AnA/Rot, Antimycin A/Rotenone; 2-DG, 2-deoxyglucose. D, DMSO + PBS (-) group; D + L, DMSO + LPS group; AX, Astaxanthin + PBS (-) group; AX + LPS, Astaxanthin + LPS group.

The effect of AX on energy shift is shown in Figure 6. Numerous studies showed that LPS stimulation induces a shift towards a glycolytic phenotype. To investigate whether AX has an effect on mitochondrial metabolism and the phenotype of AX in LPS-stimulated RAW264.7 cells, we measured key parameters of mitochondrial function by directly measuring the oxygen consumption rate (OCR) and glycolytic function by directly measuring the extracellular acidification rate (ECAR) of cells. 

For OCR, as shown in Figure 6A,B (no injection), the basal level of OCRs (OCR before oligomycin—OCR after Rot/AnA) were D: 213.32 ± 34.54 pmol/min/μg protein, D + L: 190.27 ± 35.50 pmol/min/μg protein, AX: 223.93 ± 40.14 pmol/min/μg protein and AX + L: 212.24 ± 46.83 pmol/min/μg protein. In comparison to the D group, the D + L group had a slightly lower basal OCR, although it was not significant. AX group tended to maintain a higher basal OCR compared with the D group. There were no significant differences between the AX + LPS group and other groups. Subsequently, oligomycin, as an inhibitor of ATP synthase (complex V), was injected. It impacted or decreased electron flow through the ETC, resulting in a reduction in mitochondrial respiration or OCR. This decrease in OCR was linked to cellular ATP production. Through the decreased OCR (Figure 6A,B (oligomycin+)), we found that under the condition of oligomycin injection, the D + L group showed a great reduction and significant difference to the D group in ATP-linked respiration OCR level (OCR before oligomycin—OCR after oligomycin). However, the ATP-linked respiration OCR level in the AX group and even in the AX + LPS group showed no significant differences to the D group (D: 165.20 ± 23.569 pmol/min/μg protein, D + L: 135.02 ± 21.28 pmol/min/μg protein, AX: 167.50 ± 34.02 pmol/min/μg protein and AX + L: 169.04 ± 37.07 pmol/min/μg protein). FCCP (carbonyl cyanide-4 (trifluoromethoxy) phenylhydrazone), an uncoupling agent, was the 2nd injection following oligomycin. As a result, electron flow through the ETC was uninhibited, and oxygen consumption by complex IV reached its maximum. As shown in Figure 6A,B (FCCP+), compared to the maximum respiration OCR (OCR after FCCP–OCR after Rot/AnA) of the D group (D: 532.46 ± 84.32 pmol/min/μg protein), the D + L group showed a significant decrease (D + L: 423.34 ± 79.67 pmol/min/μg protein). However, there were no significant differences between the AX group (516.89 ± 54.36 pmol/min/μg protein), the AX + L group (478.69 ± 20.04 pmol/min/μg protein), and the D group. The mixture of Rot/AnA, an inhibitor of mitochondrial complexes I and III, was the 3rd and final injection. This combination stopped mitochondrial respiration and enabled the calculation of nonmitochondrial respiration driven by processes outside of the mitochondria. As shown in Figure 6A,B (Rot/AnA+), there were no significant changes among the D group (75.05 ± 11.84 pmol/min/μg protein), the D + L group (71.10 ± 14.60 pmol/min/μg protein), the AX group (76.35 ± 6.25 pmol/min/μg protein), and the AX + L group (75.18 ± 11.52 pmol/min/μg protein). These results revealed that AX saved mitochondrial respiratory function and contributed particularly to maintaining the ATP production-related respiration and maximum respiration capacity in LPS-stimulated RAW264.7 cells.

For ECAR, the first injection was a saturated concentration of glucose (Figure 6C). The injected glucose was used by cells and metabolized into pyruvate with the production of ATP, NADH, water, and protons through the glycolytic pathway. The extrusion of protons into the surrounding medium resulted in a rapid increase in ECAR. This glucose-induced response was reported as the rate of glycolysis under basal conditions. As shown in Figure 6D (glucose+), treatment of LPS resulted in a significant increase in glycolysis rate (ECAR after glucose—ECAR before glucose, 156.70 ± 49.85 mpH/min/μg protein) compared to the D group (77.80 ± 24.30 mpH/min/μg protein). Nevertheless, the AX + LPS group (80.40 ± 33.55 mpH/min/μg protein) suppressed the upregulation of glycolysis rate, which was caused by LPS, and showed no significant differences between the D group and the AX group (89.60 ± 27.90 mpH/min/μg protein). Moreover, following the injection of oligomycin, energy production was shifted to the glycolysis process, and the subsequent increase in ECAR revealed the cellular maximal glycolytic capacity. We found that not only basal glycolysis rate, but also maximum glycolytic capacity was noticeably increased in the D + LPS group (200.06 ± 47.59 mpH/min/μg protein) compared to the D group (126.36 ± 47.59 mpH/min/μg protein). In comparison, the AX + LPS group (101.30 ± 41.80 mpH/min/μg protein) maintained similar maximum glycolytic capacity level to the D group and the AX group (140.02 ± 34.09 mpH/min/μg protein). These results demonstrated that although the stimulation of LPS increased the glycolytic rate and maximum glycolytic capacity, AX pre-treatment maintained a relatively stable glycolytic rate and maximum glycolytic capacity in the cells and remained nearly identical to unstimulated cells.

To further elaborate on the cell energy phenotype, we displayed differential metabolic parameters (Mitochondrial respiration related: Basal respiration, ATP production, Maximum respiration; Glycolytic function related: Glycolysis rate, Maximum glycolytic capacity) in Figure 6E. Apparently, compared to the D group, the D + L group showed a relative decrease in mitochondrial respiratory function accompanied by a significant increase in glycolytic capacity. However, the AX + LPS group kept a stable mitochondrial respiratory function and lower glycolysis. Overall, AX suppressed a shift from an OXPHOS phenotype towards a glycolytic phenotype in LPS-stimulated RAW264.7 cells.

## 3. Discussion

**Figure 7:**
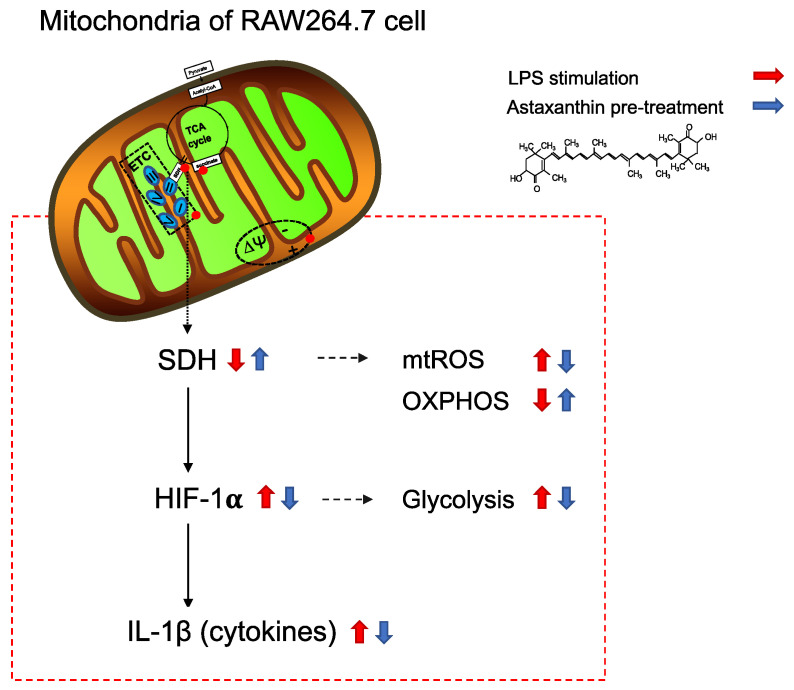
The proposed mechanisms of the immunomodulatory effect of Astaxanthin. LPS, lipopolysaccharides; TCA cycle, tricarboxylic acid cycle; ETC, electron transport chain; ∆Ψ, mitochondrial membrane potential; SDH, succinate dehydrogenase; mtROS, mitochondrial ROS; OXPHOS, oxidative phosphorylation; HIF-1α, hypoxia-inducible factor-1α; IL-β, interleukin-1β.

The novel findings of this study revealed the following: AX exerted immunomodulatory effects by reprogramming mitochondrial metabolism, AX suppressed a shift from an OXPHOS phenotype towards a glycolytic phenotype, and AX prevented HIF-1α induced IL-1β by regulating SDH activity and expression (a graphical illustration is shown in Figure 7).

Consistent with previous studies, LPS stimulation led to the release of a large number of pro-inflammatory cytokines in RAW264.7 macrophage cells [19], while AX effectively inhibited the release of these pro-inflammatory cytokines, especially in IL-1β (Figure 2A,B). Although it has been reported that AX suppressed IL-1β expression by blocking the nuclear translocation of NF-κB p65 subunit and IκBα in LPS-stimulated RAW264.7 cells [20], existing results raised our curiosity about whether AX regulates IL-1β in a unique rather than a single way, such as in an inflammasome-dependent manner or in a way regulated by mitochondria. Therefore, we investigated the effects of AX on mitochondria in the immune response.

Reactive oxygen species (ROS) production is considered the central part of the progression of inflammation [22]. It is a partial source of inflammation and a catalyst for accelerated inflammation. ROS derived from mitochondria are produced by ETC through OXPHOS and are viewed as the major source of free radicals [23]. We found that AX had an alleviative effect on mitochondrial ROS production in response to immune response signals (Figure 3A). It is possibly involved in the unique structure of AX. Particularly, the structural property of the long nonpolar conjugated double bonds and the polar groups such as keto and hydroxyl at each end of double bonds enable AX to be embedded in the cell membrane and scavenge ROS in both the inner and outer lipid layers of the cellular membrane [13,24]. Our previous studies supported that AX was more likely to accumulate in mitochondria, which membranes also have a phospholipid backbone similar to the cell membrane structure [17]. Here, we also demonstrated the possibility that AX acts on mitochondria and scavenges mitochondrial free radicals in macrophages. Likewise, the effect of AX on mitochondrial ROS was also found in gastric epithelial cells [25], alveolar epithelial cells type II [26], vascular smooth muscle cells [27], and so on.

The ETC on the mitochondria consists of 2 electron carriers (coenzyme Q [CoQ] and Cyt c) and a range of complexes (I, II, III, IV, V) in the mitochondrial inner membrane [28]. Electron leakage from at least 11 different sites within the mitochondria leads to mtROS [29], and complexes I and III are known as the major sites of ROS generation [30,31,32]. Complex I produces ROS in the mitochondrial matrix, whereas complex III can produce ROS into either the mitochondrial matrix or intermembrane space [33]. In the mitochondrial immune response, consistent with the result of Aki T and colleagues [34], we also found significant reductions in mitochondrial complexes I, II, III, and IV during LPS stimulation of macrophages and activation into the M1 phenotype (Figure 4A,B). These findings indicated the changes in mitochondrial proteins and functions as a result of LPS stimulation, as well as the phenotype changes of mitochondria under M1 macrophages. In our data, for these two primary mtROS-producing sites (complex I and III), AX improved their protein level, especially for complex I (Figure 4A,B). The complex I-derived ROS displayed an important effect on the immune system [28]. One mechanism by which complex I produces large amounts of O_2_^−^ is reverse electron transport (RET), which occurs at a high proton motive force and a reduced coenzyme Q (CoQ) pool [35]. In macrophages, it has been reported that RET-derived ROS induces pro-IL-1β production through a specific signaling pathway [36]. Moreover, the ROS produced from complex I inhibit the IL-10 expression [37] and also oxidizes mitochondrial deoxyribonucleic acid (mtDNA) and activates IL-1β [36]. These all suggest clear effects of complex I-derived ROS on the immune response and the important role of maintaining stable complex I expression for immune regulation. Although the specific sites of action of AX in mitochondrial complex I (e.g., assembly of complex I, subunit proteins of complex I, and electron transport involved in complex I) still require further investigation, we are considering that the protection of complex I by AX and stabilization of its normal expression to reduce ROS caused by complex I play a vital role in the immune response of macrophages.

SDH, also known as complex II, is a dehydrogenase on ETC as well as a member of the TCA cycle, and they are located on the matrix side of the mitochondrial inner membrane [38]. Abnormalities of SDH activity, which induce immune diseases, are usually associated with metabolic dysfunction, such as the reduced activity of SDHA and SDHB [39]. Inhibition of SDH activity impairs human T cell activation and function [40] and impairs the macrophage’s bactericidal activity in vitro [41] while maintaining the activity of SDH contributes to the anti-microbial responses [41]. In this study, LPS stimulation destroyed the SDH activity in macrophages, whereas AX completely prevented this damage (Figure 4C). The protective effect of SDH (complex II) activity seems like a part of the anti-microbial response of AX. Moreover, the reduction in SDH expression in macrophages caused by LPS leads to the accumulation of succinate in the mitochondria, and its release into the cytosol and extracellular space is perceived as an inflammation signal [42,43]. We showed that AX upregulated the decrease inSDH (especially SDHB, one subunit of SDH) mRNA and protein expression significantly, which was induced by LPS stimulation (Figure 4A,B,D). On the one hand, SDH (complex II), as a part of OXPHOS, is a site of mitochondrial superoxide production [44] and also a source of electrons that drives RET at complex I [36]. AX sustains the normal expression of SDH (complex II), which in turn prevents the release of ROS caused by SDH (complex II) dysfunction (this includes ROS leaked from SDH (complex II) itself and possibly also from complex I due to RET). On the other hand, as a member of the TCA cycle, SDH catalyzes the oxidation of succinate to fumarate [38]. There are two interruptions in the TCA cycle of M1-polarized macrophages, one of which is the SDH [11]. The interruption of SDH leads to an accumulation of succinate, which stabilizes the transcription factor hypoxia-inducible factor-1α (HIF-1α) and enhances the IL-1β production and inflammation [21,42]. Interestingly, this succinate-driven inflammation is associated with ROS [45]. Hence, the safeguard of AX on SDH (complex II) secures the function of SDH in the TCA cycle, slows down the stress from SDH (complex II) brought to complex I, and eliminates the ROS they produce through ETC, which is likely a mechanism by which AX keeps mitochondrial and macrophage homeostasis and thus slows down inflammation. 

HIF-1α, an important transcription factor, showed a vital regulatory effect on inflammatory macrophage function [46]. Macrophages normally respond to local hypoxia caused by inflammation-mediated upregulation of HIF-1α [47], while it can also be induced through stimulation, such as by LPS, under normoxic conditions [48,49]. Overexpression of HIF-1α in macrophages was found to result in the upregulation of M1 markers [50]. Conversely, the knockdown of HIF-1α decreased the M1 marker, such as IL-1β production [51]. Intriguing, the limitation of SDHB activity caused by augmented electron flux through complex II attracts HIF-1α activation in a ROS-dependent manner [52]. As described in a previous report, the accumulation of succinate stabilizes the HIF-1α and enhances the IL-1β production, resulting in inflammation, most probably by the mechanism related to ROS [21]. Analogously, the reduction in mitochondrial ROS caused by complex I can reduce the oxidative effect of LPS on mitochondrial SDH and impair HIF-1α stabilization and IL-1β expression [51]. These illustrate the important role of the collaboration of SDH, ROS, and HIF-1α in the induction of IL-1β. Based on the specific protective effect of AX on SDH, especially SDHB, and the powerful function of clearing mitochondrial free radicals, as well as the significant inhibitory effect on IL-1β, we are highly interested in whether HIF-1α is also regulated by AX in this progress. Astonishingly, AX suppressed the upregulation of HIF-1α (Figure 5A,B). Subsequently, AA5 was used to confirm the effect of AX on this progress. AA5 is one of the inhibitors of SDH, and it showed a significant inhibitory effect on SDH activity [51]. In addition, the co-treatment of LPS and AA5 inhibited *Sdhb* expression (Figure 5C), while it noticeably promoted HIF-1α (Figure 5E,F) and IL-1β (Figure 5D) expressions, compared to the treatment with AA5 alone. These results were consistent with the study of Fuhrmann DC and colleagues [51] that showed that the inhibition of SDH stabilizes HIF-1α and promotes IL-1β. However, the addition of AA5 abrogated the original downregulation of IL-1β (Figure 5D) and HIF-1α (Figure 5E,F) by AX. These results noted that the modulatory effect of AX on HIF-1α and the SDH-HIF-1α axis is involved in the IL-1β inhibitory mechanism of AX.

In LPS-activated-M1 macrophages, the metabolic shift from an OXPHOS phenotype to a glycolysis phenotype was observed [21]. This metabolic reprogramming of cells toward aerobic glycolysis, similar to cancer cell metabolism, known as the “Warburg effect,” was also found in our study (Figure 6E). Interestingly, AX suppressed this metabolic shift from OXPHOS towards glycolysis (Figure 6E). On the one hand, AX controlled mitochondrial respiration by improving mitochondrial basal, ATP-linked, and maximal OCR (Figure 6A,B). Consistent with the results of a previous report [14], the slightly elevated basal OCR in the presence of AX suggested that AX keeps mitochondrial function in a more active state (Figure 6B, basal). ATP inhibition is thought to be associated with decreased MMP [53]. LPS damaged the MMP of macrophages (Figure 3B). In subsequent respiration function tests, the addition of oligomycin impeded complex V, which made the disrupted MMP in an even more unrecoverable state. At the same time, the ATP-linked OCR was even more inhibited (Figure 6B, ATP-linked). In contrast, AX maintained a higher MMP (Figure 3B) and nearly the same ATP-linked OCR level as unstimulated macrophages, even under the stimulation of LPS (Figure 6B, ATP-linked). Simply, these regulations of AX on ATP-linked OCR probably occurred by maintaining a higher membrane potential [14]. Protection of the mitochondrial complex, smoothing of the ETC, maintaining of the MMP, and activation of mitochondria all seem to be the reasons why AX protects the mitochondria of macrophages and thus pursues a more stable state and greater maximum respiratory capacity when macrophages are stimulated (Figure 6B, maximal). On the other hand, AX inhibited the abnormal enhancement of glycolytic function, especially glycolysis rate and glycolytic capacity, during the activation of M1 macrophages, and it could be considered to make them more inclined to an unstimulated energy metabolism phenotype (Figure 6C,D). The activation and stabilization of HIF-1α decreases mitochondria OCR while increasing glycolysis [54,55]. This may be activated in both hypoxic conditions and independent of hypoxia manner such as LPS stimulation [21,56]. Increased HIF-1α is heavily involved in the expression of many important enzymes and genes related to glycolysis, such as hexokinase, phosphofructose kinase, pyruvate kinase M, GLUT1, GLUT3, and LDH-A [55]. Therefore, the downregulation of glycolysis of AX seems to be associated with the inhibitory effect on HIF-1α by AX. In summary, AX suppressed the metabolic shift from OXPHOS towards glycolysis, which may be connected to the protective effect on mitochondria and the inhibitory effect on HIF-1α by AX. We propose these hypotheses based on the available results; however, further studies are needed to investigate the exact mechanisms of how AX affects mitochondrial respiratory and glycolytic functions.

## 4. Materials and Methods

### 4.1. Cell Culture and Treatment 

RAW264.7 cells were obtained from the American Type Culture Collection (Rockville, MD, USA). They were cultured in Dulbecco’s modified Eagle medium (DMEM; D5796, Sigma-Aldrich, St. Louis, MO, USA)) supplemented with 10% fetal bovine serum (FBS; 12483020, Gibco, Grand Island, NY, USA), 10,000 units/mL penicillin-streptomycin (15140122, Gibco, Grand Island, NY, USA) and maintained at 37 °C in a 5% CO_2_. Cells (2 × 10^5^/mL) were harvested and seeded in dishes for 24 h for the attachment and until treatment.

AX was purchased from Sigma-Aldrich (SML0982, Sigma-Aldrich) and prepared in dimethyl sulfoxide (DMSO; 04524511, Fujifilm Wako Pure Chemical Corporation, Osaka, Japan) to a concentration of 4 mM stock solution. LPS from *Escherichia coli* O111:B4 was purchased from Sigma-Aldrich (L4391, Sigma-Aldrich) and prepared in PBS (-) (16623555, Fujifilm Wako Pure Chemical Corporation). Firstly, cells were seeded in cell culture dishes, and after 24 h of waiting for all cells to adhere to the wall, the medium was aspirated, and new medium with a final concentration of 10 μM AX or new medium with the same amount of DMSO was added (as a control group) for 24 h. Then, the medium was changed, and a new medium with 1 μg/mL LPS or PBS (-) was added for the indicated times.

### 4.2. Quantitative Real-Time Polymerase Chain Reaction (qRT-PCR)

Total RNA was extracted from cells using an acid guanidinium thiocyanate-phenol-chloroform mixture (ISOGENTM; Nippon Gene, Tokyo, Japan). Reverse transcription for cDNA synthesis and qRT-PCR analysis was performed with the appropriate primers and SYBR Green dye using a real-time PCR system (ABI Real-Time PCR Detection System; Applied Biosystems, Foster City, CA, USA), as described previously [17]. The mRNA levels were normalized to the housekeeping gene *18S* ribosomal RNA. The oligonucleotide primers used for PCR are as follows: 5′-CTCATCTGGGATCCTCTCCA-3′ and 5′-GGGTCCGTCAACTTCAAAG-A-3′ for mouse *Il-1b*; 5′-GGAGGGCAAGCAACAGTATC-3′ and 5′ and 5′-CTTGTCTCCGTTCCACCAGT-3′ for *Sdhb*; 5′-CCATCCAATCGGTAGTAGCG-3′ and 5′-GTAACCCGTTG-AACCCCATT-3′ for mouse *18S*.

### 4.3. Enzyme-Linked Immunosorbent Assay (ELISA)

For the detection of released IL-1β, ELISA (R&D Systems, Inc (Minneapolis, MN, USA)) was performed following the manufacturer’s instructions. Briefly, cell culture supernatants were collected and centrifuged at 12,000 rpm at 4 °C for 5 min, and supernatants were acquired for detection. The collected supernatants were added into microplate wells which were coated with diluent assay RD1N (R&D Systems, 895488) for 2 h incubation at room temperature (RT). Free unbound reactants were removed by aspirating and washing. After the last wash, the remaining wash buffer was removed, followed by incubation with mouse IL-1β conjugate (R&D Systems, 893830) for 2 h, RT. The preceding washing step was repeated, and the substrate solution was added and incubated (mixture of color A reagent and color B reagent with a ratio of 1:1) for 30 min, RT, protected from light. The absorbance at 450 nm was measured by a microplate reader (BioTek Cytation 3, Winooski, VT, USA).

### 4.4. Mitochondrial Superoxide Levels Measurement

The superoxide production from mitochondria was detected using MitoSOXTM Red fluorescent probe, Invitrogen, as described by the protocol. Cells were harvested and seeded in a black 96-well plate with a density of 5 × 10^4^ cells/well, AX (10 μM)/DMSO were treated for 24 h, then LPS (1 μg/mL)-stimulated for 6 h with fresh media. After that, cells were washed by warm HBSS and incubated with 5 μM MitoSOX reagent (M36008, Thermo Fisher Scientific, Eugene, Ore., USA) for 30 min at 37 °C, protected from light. Fluorescence was recorded using a microplate reader (BioTek Cytation 3, Winooski, VT, USA) at excitation and emission wavelengths of 490 and 595 nm, respectively.

### 4.5. Mitochondrial Membrane Potential (MMP) Measurement

For MMP measurement, the pre-treatment of AX and stimulation of LPS were consistent with mitochondrial superoxide production measurement. After that, cells were washed with PBS and incubated with 1.5 μM JC-1 dye (Thermo Fisher Scientific) for 30 min at 37 °C, protected from light. Fluorescence was recorded using a microplate reader (BioTek Cytation 3) at 550 nm excitation/600 nm emission and 485 nm excitation/535 nm emission wavelengths, respectively.

### 4.6. Protein Extraction and Immunoblotting

Protein samples from cells were prepared in Lysis buffer containing 50 mM Tris-HCl, pH 7.5, 150 mM NaCl, 1% Triton X-100, 5 mM EDTA, 10 mM NaF, 2 mM Na_3_VO_4_, and a protease inhibitor cocktail tablet without EDTA (Roche Diagnostics, Indianapolis, IN, USA) and homogenized using a sonicator. The Pierce BCA assay (Pierce, Rochford, IL, USA) was used to quantify proteins. Protein samples were combined with 4 X sample buffer (250 mM Tris-HCl, 8% SDS, 40% glycerol, 8% beta-mercaptoethanol, and 0.02% bromophenol blue) and subjected to SDS-PAGE. The proteins were transferred onto a polyvinylidene difluoride (PVDF) membrane and probed with primary antibodies according to the manufacturer’s instructions. Anti-Total OXPHOS, anti-HIF-1α (NB100-449, Novus, USA), and anti-β-actin (GTX629630, AC-15, Gene Tex, Irvine, CA, USA) were used. Donkey anti-rabbit IgG (NA934V, GE Healthcare, Little Chalfont, UK) at 1:5000 and sheep anti-mouse IgG (NAX931, GE Healthcare) at 1:5000 was used as the secondary antibodies. Membranes were developed using ImmunoStar^®^ Zeta Western blotting detection reagents (Fujifilm Wako Pure Chemical Corporation, Osaka, Japan). Immunocomplexes on the membrane were analyzed by Image J software (National Institutes of Health, Bethesda, MD, USA).

### 4.7. Succinate Dehydrogenase (SDH) Activity Assay

The activity of SDH was detected by using a colorimetric method according to the manufacturer’s guidelines. Briefly, 1 × 10^6^ cells/sample were homogenized with 100 μL ice-cold SDH assay buffer, then centrifuged at 10,000 g for 5 min, 4 °C. The supernatants were collected in new tubes. The supernatant was diluted with SDH assay buffer, then 50 μL dilution of supernatant was loaded in each well of the 96-well plate. Subsequently, 50 μL SDH reaction mix (SDH assay buffer 46 μL, SDH probe 2 μL, and SDH substrate mix 2 μL) was added to each well to make a final volume of 100 μL per well. The absorbance was measured at a wavelength of 600 nm for 30 min with an interval every 3 min.

### 4.8. Extracellular Flux Analysis

The oxygen consumption rates (OCRs), and the extracellular acidification rates (ECAR) were measured using the XFe96 Extracellular Flux Analyzer (Seahorse Bioscience, North Billerica, MA, USA), modified as the previous report [57]. Cells were plated at 4 × 10^4^ cells/well on a 96-well Seahorse cell culture plate and incubated overnight in a growth medium. Then, cells were treated with AX (10 μM) for 24 h and stimulated with LPS (1 μg/mL) for 3 h. For hydrating the cartridge, a sensor cartridge was prepared 24 h before Seahorse XFe96 operation. The sensor cartridge was lowered onto the utility plate and submerged in the sterilization water (200 μL/well) in a non-CO_2_ incubator at 37 °C overnight. 

For OCRs detection, on the operation day, after 3 h stimulation of LPS, the cell culture medium was removed from the cells and replaced with XF DMEM medium (supplemented with 10 mM glucose, 1 mM pyruvate, and 2 mM L-glutamine). The cell culture plate was sited in a non-CO_2_ incubator at 37 °C for 1 h. Additionally, the sterilization water was removed and discarded from the utility plate, then replaced with the pre-warmed XF Calibrant (200 μL/well). The assembled sensor cartridge with the utility plate was placed in a non-CO_2_ incubator at 37 °C for 1 h prior to loading the injection ports of the sensor cartridge. The OCRs were detected under basal conditions, and after the application of 1.5 μM oligomycin to measure the ATP-related oxygen consumption, 2 μM carbonyl cyanide 4-trifluoromethoxy phenylhydrazone (FCCP) to measure the maximum respiratory capacity, and 1 μM rotenone + 1 μM antimycin A to measure the non-mitochondrial oxygen consumption. 

For ECARs detection, on the operation day, after 3 h stimulation of LPS, the cell culture medium was removed from the cells and replaced with XF DMEM medium (supplemented with 2 mM L-glutamine). The cell culture plate was sited in a non-CO_2_ incubator at 37 °C for 1 h. Additionally, the sterilization water was removed and discarded from the utility plate, then replaced with the pre-warmed XF Calibrant (200 μL/well). The assembled sensor cartridge with a utility plate was placed in a non-CO_2_ incubator at 37 °C for 1 h prior to loading the injection ports of the sensor cartridge. The ECARs were detected under basal conditions and after the application of 10 mM glucose to measure the glycolysis, 1 μM oligomycin to measure the glycolytic capacity, and 50 mM 2-deoxy-glucose (2-DG) to measure the glycolytic reserve.

After completion of the OCR and ECAR detection, the cells were washed with PBS once and lysed in a lysis buffer. The lysates were centrifuged to obtain the protein supernatants, and the protein concentration was detected by BCA assay. The OCR and ECAR values were normalized by the protein concentration and presented as pmol/min/μg protein and mpH/min/μg protein.

### 4.9. Statistical Analysis

All data were analyzed by one-way analysis of variance (ANOVA), using IBM SPSS statistics version 26.0 software (Armonk, NY, USA), followed by the Tukey or Tukey Kramer (for unequal number) test for individual differences between groups. *p*-values < 0.05 were considered to indicate significant differences.

## 5. Conclusions

We have identified an important regulatory role of AX on the mutation of mitochondrial SDH caused by external stimuli during the polarization of macrophages toward the M1 type. AX inhibited the stabilization of HIF-1α and IL-1β by regulating SDH. Furthermore, we have demonstrated that AX suppressed the energy shift from an OXPHOS phenotype to a glycolysis phenotype, which was closely related to the protective effect on mitochondria and the inhibitory effect on HIF-1α by AX. These findings revealed important effects of astaxanthin on mitochondrial enzymes as well as on mitochondrial energy metabolism in the immune response. In addition, it was suggested that astaxanthin might play an important role in other diseases caused by SDH mutation and metabolic disorders.

## Data Availability

Not applicable.

## Abbreviations

AA5	Atpenin A5
AnA	antimycinA
ATP	adenosine triphosphate
AX	astaxanthin
BCA	bicinchoninic acid
DMEM	Dulbecco’s modified Eagle medium
DMSO	dimethyl sulfoxide
DNA	deoxyribonucleic acid
ECAR	extracellular acidification rate
EDTA	ethylenediaminetetraacetic acid
ELISA	enzyme-linked immunosorbent assay
ETC	electron transport chain
FBS	fetal bovine serum
FCCP	carbonyl cyanide 4-trifluoromethoxy phenylhydrazone
HBSS	Hank’s balanced salt solutions
HIF-1α	hypoxia-inducible factor-1α
IL	interleukin
iNOS	inducible nitric oxide synthase
LPS	lipopolysaccharides
MCP	monocyte chemoattractant protein
MMP	mitochondrial membrane potential
mtDNA	mitochondrial DNA
mtROS	mitochondrial reactive oxygen species
NADH	nicotinamide adenine dinucleotide
OCR	oxygen consumption rate
OXPHOS	oxidative phosphorylation
PBS	phosphate buffered saline
qRT-PCR	quantitative real-time polymerase chain reaction
ROS	reactive oxygen species
Rot	rotenone
SDH	succinate dehydrogenase
Sdhb	succinate dehydrogenase complex, subunit B
TCA	tricarboxylic acid
TNF	tumor necrosis factor
2-DG	2-deoxy-glucose

## References

[1] Shapouri-moghaddam A., Mohammandian S., Vazini H., Taghadosi M., Esmaeili S.A., Mardani F., Seifi B., Mohammadi A., Afshari J.T., Sahebkar A. (2018). Macrophage plasticity, polarization, and function in health and disease. J. Cell Physiol..

[2] Chen Y., Hu M., Wang L., Chen W. (2020). Macrophage M1/M2 polarization. Eur. J. Pharmacol..

[3] Sica A., Mantovani A. (2012). Macrophage plasticity and polarization: In vivo veritas. J. Clin. Investig..

[4] Mills E.L., Kelly B., O’Neil L.A.J. (2017). Mitochondria are the powerhouses of immunity. Nat. Immunol..

[5] Weinberg S.E., Sena L.A., Chandel N.S. (2015). Mitochondria in the regulation of innate and adaptive immunity. Immunity.

[6] Langston P.K., Shibata M., Horng T. (2017). Metabolism supports macrophage activation. Front Immunol..

[7] Ganeshan K., Chawla A. (2014). Metabolic regulation of immune responses. Annu. Rev. Immunol..

[8] EI-Kasmi K.C., Stenmark K.R. (2015). Contribution of metabolic reprogramming to macrophage plasticity and function. Semin. Immunol..

[9] Van den Bossche J., Baardman J., Otto N.A., van der Velden S., Neele A.E., van den Berg S.M., Luque-Martin R., Chen H.-J., Boshuizen M.C.S., Ahmed M. (2016). Mitochondrial dysfunction prevents repolarization of inflammatory macrophages. Cell Rep..

[10] Wang Y., Li N., Zhang X., Horng T. (2021). Mitochondrial metabolism regulates macrophage biology. J. Biol. Chem..

[11] Mills E.L., O’Neil L.A. (2016). Reprogramming mitochondrial metabolism in macrophages as an anti-inflammatory signal. Eur. J. Immunol..

[12] Yuan J.P., Peng J., Yin K., Wang J.H. (2011). Potential health-promoting effects of astaxanthin: A high-value carotenoid mostly from microalgae. Mol. Nutr. Food Res..

[13] Ambati R.R., Phang S.M., Ravi S., Aswathanarayana R.G. (2014). Astaxanthin: Sources, extraction, stability, biological activities and its commercial applications—A review. Mar. Drugs.

[14] Wolf A.M., Asoh S., Hiranuma H., Ohsawa I., Iio K., Satou A., Ishikura M., Ohta S. (2010). Astaxanthin Protects Mitochondrial Redox State and Functional Integrity Against Oxidative Stress. J. Nutr. Biochem..

[15] Zhang Z.W., Xu X.C., Liu T., Yuan S. (2016). Mitochondrion-Permeable Antioxidants to Treat ROS-Burst-Mediated Acute Diseases. Oxid. Med. Cell Longev..

[16] Kuroki T., Ikeda S., Okada T., Maoka T., Kitamura A., Sugimoto M., Kume S. (2013). Astaxanthin ameliorates heat stress-induced impairment of blastocyst development in vitro: --astaxanthin colocalization with and action on mitochondria--. J. Assist. Reprod. Genet..

[17] Sun L., Miyaji N., Yang M., Mills E.M., Taniyama S., Uchida T., Nikawa T., Li J., Shi J., Tachibana K. (2021). Astaxanthin prevents atrophy in slow muscle fibers by inhibiting mitochondrial reactive oxygen species via a mitochondria-mediated apoptosis pathway. Nutrients.

[18] Chang M.X., Xiong F. (2020). Astaxanthin and its effects in inflammatory responses and inflammation-associated diseases: Recent advances and future directions. Molecules.

[19] Lee S., Bai S., Lee K., Namkoong S., Na H., Ha K., Han J., Yim S., Chang K., Kwon Y. (2003). Astaxanthin inhibits nitric oxide production and inflammatory gene expression by suppressing I(kappa)B kinase-dependent NF-kappaB activation. Mol. Cells.

[20] Farruggia C., Kim M.B., Bae M., Lee Y., Pham T.X., Yang Y., Han M.J., Park Y.K., Lee J.Y. (2018). Astaxanthin exerts anti-inflammatory and antioxidant effects in macrophages in NRF2-dependent and independent manners. J. Nutr. Biochem..

[21] Tannahill G.M., Curtis A.M., Adamik J., Palsson-McDermott E.M., McGettrick A.F., Goel G., Frezza C., Bernard N.J., Kelly B., Foley N.H. (2013). Succinate is an inflammatory signal that induces IL-1β through HIF-1α. Nature.

[22] Mittal M., Siddiqui M.R., Tran K., Reddy S.P., Malik A.B. (2014). Reactive oxygen species in inflammation and tissue injury. Antioxid. Redox Signal.

[23] Ravera S., Bartolucci M., Cuccarolo P., Litamè E., Illarcio M., Calzia D., Degan P., Morelli A., Panfoli I. (2015). Oxidative stress in myelin sheath: The other face of the extramitochondrial oxidative phosphorylation ability. Free Radic. Res..

[24] Higuera-Ciapara I., Félix-Valenzuela L., Goycoolea F.M. (2006). Astaxanthin: A review of its chemistry and applications. Crit. Rev. Food Sci. Nutr..

[25] Kim S.H., Lim J.W., Kim H. (2018). Astaxanthin Inhibits Mitochondrial Dysfunction and Interleukin-8 Expression in Helicobacter pylori-Infected Gastric Epithelial Cells. Nutrients.

[26] Song X., Wang B., Lin S., Jing L., Mao C., Xu P., Lv C., Liu W., Zuo J. (2014). Astaxanthin inhibits apoptosis in alveolar epithelial cells type II in vivo and in vitro through the ROS-dependent mitochondrial signalling pathway. J. Cell Mol. Med..

[27] Chen Y., Li S., Guo Y., Yu H., Bao Y., Xin X., Yang H., Ni X., Wu N., Jia D. (2020). Astaxanthin Attenuates Hypertensive Vascular Remodeling by Protecting Vascular Smooth Muscle Cells from Oxidative Stress-Induced Mitochondrial Dysfunction. Oxid. Med. Cell Longev..

[28] Yin M., O’Neill L.A.J. (2021). The role of the electron transport chain in immunity. FASEB J..

[29] Brand M.D. (2016). Mitochondrial generation of superoxide and hydrogen peroxide as the source of mitochondrial redox signaling. Free Radic. Biol. Med..

[30] Goncalves R.L., Quinlan C.L., Perevoshchikova I.V., Hey-Mogensen M., Brand M.D. (2015). Sites of superoxide and hydrogen peroxide production by muscle mitochondria assessed ex vivo under conditions mimicking rest and exercise. J. Biol. Chem..

[31] Chouchani E.T., Pell V.R., Gaude E., Aksentijević D., Sundier S.Y., Robb E.L., Logan A., Nadtochiy S.M., Ord E.N.J., Smith A.C. (2014). Ischaemic accumulation of succinate controls reperfusion injury through mitochondrial ROS. Nature.

[32] Scialò F., Sriram A., Fernández-Ayala D., Gubina N., Lõhmus M., Nelson G., Logan A., Cooper H.M., Navas P., Enríquez J.A. (2016). Mitochondrial ROS Produced via Reverse Electron Transport Extend Animal Lifespan. Cell. Metab..

[33] Scialò F., Fernández-Ayala D.J., Sanz A. (2017). Role of Mitochondrial Reverse Electron Transport in ROS Signaling: Potential Roles in Health and Disease. Front. Physiol..

[34] Aki T., Funakoshi T., Noritake K., Unuma K., Uemura K. (2020). Extracellular glucose is crucially involved in the fate decision of LPS-stimulated RAW264.7 murine macrophage cells. Sci. Rep..

[35] Murphy M.P. (2009). How mitochondria produce reactive oxygen species. Biochem. J..

[36] Mills E.L., Kelly B., Logan A., Costa A.S., Varma M., Bryant C., Tourlomousis P., Däbritz J.H.M., Gottlieb E., Latorre I. (2016). Succinate Dehydrogenase Supports Metabolic Repurposing of Mitochondria to Drive Inflammatory Macrophages. Cell.

[37] Kelly B., Tannahill G.M., Murphy M.P., O’Neill L.A. (2015). Metformin Inhibits the Production of Reactive Oxygen Species from NADH: Ubiquinone Oxidoreductase to Limit Induction of Interleukin-1β (IL-1β) and Boosts Interleukin-10 (IL-10) in Lipopolysaccharide (LPS)-activated Macrophages. J. Biol. Chem..

[38] Hederstedt L., Rutberg L. (1981). Succinate dehydrogenase--a comparative review. Microbiol. Rev..

[39] Moreno C., Santos R.M., Burns R., Zhang W.C. (2020). Succinate Dehydrogenase and Ribonucleic Acid Networks in Cancer and Other Diseases. Cancers.

[40] Nastasi C., Willerlev-Olsen A., Dalhoff K., Ford S.L., Gadsbøll A.Ø., Buus T.B., Gluud M., Danielsen M., Litman T., Bonefeld C.M. (2021). Inhibition of succinate dehydrogenase activity impairs human T cell activation and function. Sci. Rep..

[41] Garaude J., Acín-Pérez R., Martínez-Cano S., Enamorado M., Ugolini M., Nistal-Villán E., Hervás-Stubbs S., Pelegrín P., Sander L.E., Enríquez J.A. (2016). Mitochondrial respiratory-chain adaptations in macrophages contribute to antibacterial host defense. Nat. Immunol..

[42] Mills E., O’Neill L.A. (2014). Succinate: A metabolic signal in inflammation. Trends Cell Biol..

[43] Murphy M.P., O’Neill L.A.J. (2018). Krebs Cycle Reimagined: The Emerging Roles of Succinate and Itaconate as Signal Transducers. Cell.

[44] Brand M.D. (2010). The sites and topology of mitochondrial superoxide production. Exp. Gerontol..

[45] Ryan D.G., O’Neill L.A.J. (2020). Krebs Cycle Reborn in Macrophage Immunometabolism. Annu. Rev. Immunol..

[46] McGettrick A.F., O’Neill L.A.J. (2020). The Role of HIF in Immunity and Inflammation. Cell Metab..

[47] Murdoch C., Muthana M., Lewis C.E. (2005). Hypoxia regulates macrophage functions in inflammation. J. Immunol..

[48] Blouin C.C., Pagé E.L., Soucy G.M., Richard D.E. (2004). Hypoxic gene activation by lipopolysaccharide in macrophages: Implication of hypoxia-inducible factor 1alpha. Blood.

[49] Mi Z., Rapisarda A., Taylor L., Brooks A., Creighton-Gutteridge M., Melillo G., Varesio L. (2008). Synergystic induction of HIF-1alpha transcriptional activity by hypoxia and lipopolysaccharide in macrophages. Cell Cycle.

[50] Takeda N., O’Dea E.L., Doedens A., Kim J.W., Weidemann A., Stockmann C., Asagiri M., Simon M.C., Hoffmann A., Johnson R.S. (2010). Differential activation and antagonistic function of HIF-{alpha} isoforms in macrophages are essential for NO homeostasis. Genes Dev..

[51] Fuhrmann D.C., Wittig I., Brüne B. (2019). TMEM126B deficiency reduces mitochondrial SDH oxidation by LPS, attenuating HIF-1α stabilization and IL-1β expression. Redox Biol..

[52] Guzy R.D., Sharma B., Bell E., Chandel N.S., Schumacker P.T. (2008). Loss of the SdhB, but Not the SdhA, subunit of complex II triggers reactive oxygen species-dependent hypoxia-inducible factor activation and tumorigenesis. Mol. Cell. Biol..

[53] Ainscow E.K., Brand M.D. (1999). Internal regulation of ATP turnover, glycolysis and oxidative phosphorylation in rat hepatocytes. Eur. J. Biochem..

[54] Selak M.A., Armour S.M., MacKenzie E.D., Boulahbel H., Watson D.G., Mansfield K.D., Pan Y., Simon M.C., Thompson C.B., Gottlieb E. (2005). Succinate links TCA cycle dysfunction to oncogenesis by inhibiting HIF-alpha prolyl hydroxylase. Cancer Cell.

[55] Taylor C.T., Scholz C.C. (2022). The effect of HIF on metabolism and immunity. Nat. Rev. Nephrol..

[56] Corcoran S.E., O’Neill L.A. (2016). HIF1α and metabolic reprogramming in inflammation. J. Clin. Investig..

[57] Kim S.E., Mori R., Komatsu T., Chiba T., Hayashi H., Park S., Sugawa M.D., Dencher N.A., Shimokawa I. (2015). Upregulation of cytochrome c oxidase subunit 6b1 (Cox6b1) and formation of mitochondrial supercomplexes: Implication of Cox6b1 in the effect of calorie restriction. Age.

